# Immune activation and regulation mediated by immune cell-derived EVs (iEVs)

**DOI:** 10.1042/EBC20253005

**Published:** 2025-05-20

**Authors:** Fei Wang, Xinye Wang, Xuehao Zhang, Mengying Hu

**Affiliations:** Division of Pharmaceutics and Pharmacology, College of Pharmacy, The Ohio State University, Columbus, OH 43210, U.S.A.

**Keywords:** biotechnology, immunology, immunomodulation, nanoparticles, nanotechnology

## Abstract

Extracellular vesicles (EVs), secreted by all cellular organisms, are pivotal mediators of intercellular communication. By transporting biologically active cargos such as proteins, lipids, and nucleic acids, EVs facilitate transfer of molecular signals, effectively reflecting the characteristics of their parent cells. Immune cellderived EVs (iEVs) play a crucial role in the activation and regulation of both adaptive and innate immune responses. In the context of immune activation, iEVs drive immune cell development and activation, as well as enhance antigen presentation through both direct and cross-dressing mechanisms. Furthermore, iEVs act as signaling entities within immunological synapses, significantly amplifying immune response efficiency. In immune regulation, iEVs modulate the expression of immune checkpoint (IC) molecules and sustain immune homeostasis by transporting immunosuppressive cytokines and microRNAs, thereby mitigating excessive immune reactions. Nevertheless, the mechanistic underpinnings of iEV-mediated immune cell activation, antigen presentation, and immunoregulation remain inadequately explored. This review provides a comprehensive overview of the functions of iEVs from diverse immune cell origins and underlying mechanisms. It also examines cutting-edge engineering strategies targeting iEVs and their parent cells, while discussing their promising applications in oncology and immune-related diseases. These insights lay the foundation for the rational development of next-generation immunotherapies. While promising, the clinical translation of iEVs is hindered by low yield, high batch-to-batch variability, and insufficient targeting efficiency. The final section discusses key challenges and potential solutions.

## Introduction

Extracellular vesicles (EVs) are naturally occurring nano- to micron-sized vesicles (30–5000 nm) secreted by all cellular organisms. Based on their biogenesis and particle size, EVs are classified into non-membranous exomeres (~35 nm) and lipid bilayer-enclosed vesicles, including exosomes (50–150 nm), microvesicles (200–1000 nm), and apoptotic bodies (1000–5000 nm) [1-3]. The biogenesis of most EV subtypes involves the dynamic interaction of various membrane-bound subcellular organelles, such as endosomes, endoplasmic reticulum, Golgi apparatus, and nucleus. Therefore, EVs carry a diverse cargo of proteins, lipids, and nucleic acids reflective of their parent cells [4,5]. This unique molecular repertoire underscores their role as a bona fide mechanism for intercellular communication, facilitating the exchange of biomolecules and signals.

The recent success of immunotherapy across diverse disease settings has stimulated increased interest in the immune system, a highly intricate network of diverse cell types that utilizes both direct cell–cell interactions and contact-independent communication via soluble factors and EVs. Immune cell-derived EVs (iEVs), representing miniature of immune cells, have the potential to both activate and regulate adaptive and innate immunity through mechanisms such as stimulating or suppressing immune cell development and activity, modulating cytokine production, and influencing antigen presentation [6,7]. Elucidating the roles and molecular mechanisms underlying iEV-mediated immune activation and regulation offers critical insights for the rational design of next-generation immunotherapies. This review provides a comprehensive overview and the latest insights into the role of iEVs in immune activation and regulation, along with their engineering strategies, associated challenges, and potential opportunities for clinical translation. Additionally, the therapeutic potential of iEVs as novel immunotherapeutic strategies is explored within each subsection.

## Immune activation

### iEV-driven B cell development, activation, and humoral immune response

CD24+ EVs, predominantly secreted by immature B lymphocytes, play a vital role in the intracellular transfer of CD24, a cell surface marker that functions as a quality control mechanism to eliminate autoreactive B cell lineages [8]. This process influences B cell development and selection throughout critical stages of maturation [7,9]. In addition to CD24, B cell receptors (BCRs) are incorporated into B cell-derived EVs and can be transferred to recipient B cells, thereby expanding BCR repertoire and enhancing their capacity to recognize and respond to new antigens [9,10].

Beyond EVs secreted by B cells themselves, CD4+ T cell-derived EVs have been shown to deliver specific microRNAs (miRNAs), such as miR-20a-5p, miR-25–3p, and miR-155–3p, to B cells within germinal centers. These miRNAs regulate essential genes such as PTEN, promoting B cell survival, proliferation, and class-switch recombination, processes crucial for effective antibody production and memory B cell development [11]. Recent findings further underscore the role of B cell-derived EVs in humoral immunity, as they carry antigen-specific immunoglobulin G (IgG) that allows to neutralize virus infection [12]. Collectively, iEVs play an important role in B cell development, activation, and humoral immune responses. However, due to the inherent fragility of B cells, particularly during *in vitro* culture, studies investigating B cell-derived EVs and the underlying mechanisms of iEV-mediated B cell activation remain limited, presenting significant opportunities for future research.

### T cell development, activation, and immune boosting effect of T cell-derived EVs

Before migrating to secondary lymphoid organs, T lymphocyte precursors undergo stringent negative and positive selection processes in the thymus to ensure the expression of T cell receptors that are major histocompatibility complex (MHC) restricted but tolerant to self-antigens, a prerequisite for their maturation into functional T cells [13]. Thymic epithelial cell-derived EVs play a critical role in these selection processes by transporting tissue-restricted antigens (TRAs), a critical source of self-antigens, to thymic conventional dendritic cells (DCs). This EV-mediated TRA transfer enables DCs to present an enriched repertoire of self-antigens to developing thymocytes, facilitating the elimination of thymocytes reactive to TRAs and thereby reducing the risk of autoimmunity [13,14]. In addition to the negative selection, thymic EVs contribute to the maturation of single-positive thymocytes (CD4+ or CD8+) and their subsequent egress from the thymus. These vesicles deliver key proteins involved in thymocyte maturation and migration, including sphingosine-1-phosphate receptor 1, Rho GDP-dissociation inhibitor 1, dedicator of cytokinesis protein 2, and p21-activated kinase 2 [15-17].

Mature T cells are activated upon recognizing MHC-antigen peptide complexes presented by activated DCs. Beyond direct antigen presentation by DCs, DC-derived EVs (DCEVs) have been shown to carry costimulatory molecules and intact MHC-antigen complexes, enhancing T cell activation [18]. These mechanisms are explored in greater detail in subsequent sections.

The immunostimulatory effects of activated T cell-derived EVs have been widely recorded. For instance, mitochondrial DNA can be sorted into EVs secreted by T cells activated with phorbol myristate acetate and ionomycin, which trigger the stimulator of interferon genes (STING) signaling pathway in DCs, providing these cells with protection against pathogenic infections [19]. Similarly, activated CD4+ T cells secrete IFNγ + EVs to enhance STING activation in tumor-associated macrophages, reprogramming them toward a pro-inflammatory phenotype and eventually amplifying the efficacy of STING-based cancer immunotherapies [20]. In another study focusing on the anti-tumor potential of T cell-derived EVs, miRNAs such as miR-25–3p, miR-155–5p, miR-215–5p, and miR-375 were found to be enriched in EVs secreted from IL-2-stimulated CD4+ T cells [21]. These EV-miRNAs increased the anti-tumor response of CD8+ T cells by enhancing their proliferation and activity without affecting regulatory T cells. On the other hand, activated T cell-derived EVs also harbor tRNA fragments (tRFs), which are generated through endonucleolytic cleavage of tRNAs. These tRFs are more abundant than miRNAs in T cell-derived EVs and may confer immunosuppressive signals to recipient cells. Indeed, blocking EV biogenesis and retaining activation-induced tRFs within T cells can suppress their further activation [22]. Therefore, a deeper understanding of the dual roles of T cell-derived EVs is critical for harnessing T cell-derived EVs in therapeutic applications.

### Multifaceted immune activation by natural killer cell-derived EVs

Natural killer (NK) cells are innate lymphoid cells capable of killing infected or malignantly transformed cells and secreting pro-inflammatory cytokines. They also secrete CD56+ EVs, which exhibit immune-stimulatory functions and exert direct cytotoxic effects. For instance, NK cell-derived EVs (NKEVs) carry various cytotoxic proteins, including perforin, granzyme A, and granulysin, inducing cancer cell death through caspase-dependent, caspase-independent, and endoplasmic reticulum stress-related pathways [23]. In addition, NKEVs contain immune-boosting molecules such as IFN-γ, TNF-α, IL-10, MHC-I, and MHC-II, shaping an inflammatory microenvironment and enhancing the immunogenicity of recipient cells [24]. NKEVs were also reported to stimulate immune cells by promoting T cell activation and proliferation and inducing macrophage polarization towards a pro-inflammatory (M1) phenotype [25]. As a reinforcing feedback mechanism, NKEVs can up-regulate the expression of natural cytotoxicity receptors on NK cells, thereby improving their cytotoxicity against tumor cells. Moreover, NKEVs exhibit resistance to immunosuppression. For instance, NKEVs carrying miR-186 once being taken up by NK cells, down-regulate TGF-β receptors (TGFBR1/2), thereby reducing the inhibitory effects of TGF-β on the recognition and clearance of tumor cells [26]. Other studies have found that the addition of NKEV reduces PD-1 expression on CD3+ T cells even in the presence of TGF-β and IL-10 [27].

### iEV-mediated antigen presentation

Antigen presentation is a fundamental immunological process, where proteomic changes, indicating the presence of infections or oncogenic mutations, are processed and loaded onto MHC molecules. Since T lymphocytes recognize antigens exclusively in the context of MHC molecules, antigen presentation serves as the cornerstone of T cell-mediated immunosurveillance [28]. EVs secreted by antigen-presenting cells (APCs), including DCs, B cells, and macrophages, are enriched with MHC class I and II molecules, co-stimulatory proteins, and specific antigens. This unique molecule composition highlights their potential to directly engage T cells or facilitate intercellular transfer of MHC-antigen complexes between cells, hence amplifying antigen presentation and immune activation [29,30].

### Direct antigen presentation to T cells

DCEVs have been reported to maintain biologically functional MHC–antigen complex, enabling direct antigen presentation to CD4+ and CD8+ T cells. This process supports T cell activation and proliferation, depending on the type of MHC molecule involved [31,32]. For instance, DCs treated with tumor-associated antigens (TAAs) can secrete EVs that preserve the antigen-presenting capability of their parental DCs, effectively activating TAA-specific T cells and inducing TAA-specific immune responses [33]. Notably, EVs secreted from mature DCs exhibit a 100-fold higher efficacy in antigen presentation compared with those from immature DCs, resulting in stronger T cell stimulation both *in vitro* and *in vivo* [34]. Leveraging their enriched surface expression of MHC-I molecules, DCEVs have been engineered to carry peptides from various viruses, including respiratory syncytial virus, Epstein-Barr virus, cytomegalovirus, and influenza virus, successfully eliciting IFN-γ production by virus-specific CD8+ T cells *in vitro* [35]. However, despite their capacity for antigen presentation, DCEVs generally show lower T cell stimulation efficacy compared with their parental DCs, which may explain the failure of virus peptide-engineered EVs in priming antigen-specific CD8+ T cell responses *in vivo* [36].

In addition to DCs, activated platelet-derived EVs (aPEVs) demonstrate potent antigen presentation capabilities. These vesicles are enriched with functional proteasomes, MHC-I, and costimulatory molecules such as CD40L and OX40L [37]. aPEVs can process antigens facilitated by proteasome, load antigenic peptides onto MHC-I molecules, and successfully induce antigen-specific CD8+ T cell proliferation. Notably, aPEVs preferentially distribute to lymphoid organs, including the spleen, lymph nodes, and bone marrow, while exhibiting lower level accumulation in the liver and lungs [37]. A more recent study highlighted the diverse immunomodulatory profiles of aPEVs, determined by the specific platelet receptors activated, such as glycoprotein VI, C-type lectin-like receptor 2, or thrombin-collagen receptors. These findings suggest that while aPEVs hold significant promise as a novel vaccine platform, precise receptor-mediated activation is essential to ensure antigen presentation without triggering immune suppression [38].

### EV-mediated cross-dressing driven antigen presentation

EV-mediated cross-dressing refers to the transfer of preformed MHC–antigen complexes between APCs or from donor APCs to recipient tumor cells, enabling these recipient cells to present antigens without requiring internal antigen processing [39]. In a canonical antigen presentation pathway, DCs infected with pathogens or exposed to foreign antigenic proteins degrade these proteins via proteasomes in the cytosol [40]. The resulting peptides are transported into the endoplasmic reticulum, where they are loaded onto MHC-I molecules. The assembled MHC–antigen complexes are subsequently presented on the cell surface for T cell engagement [40]. Beyond this classic intracellular antigen processing and presentation machinery, EV-mediated intercellular transfer of MHC–antigen complexes between DCs provides an alternative route to amplify the antigen-presenting capacity of DCs. For instance, EVs derived from virus-infected APCs can transfer viral MHC–antigen complexes to uninfected APCs, thereby enhancing antiviral responses and the proliferation of memory CD8+ T cells. Nevertheless, cross-dressed APCs generally exhibit lower antigen presentation efficacy compared with those undergoing intracellular antigen processing and presentation [41].

A critical immune evasion strategy in tumors involves the down-regulation or loss of MHC molecules, which impairs their ability to present antigens and diminishes immune surveillance [42]. Interestingly, tumor cells can re-acquire antigen presentation-related molecules, such as MHC and CD86, following treatment with DCEVs [43]. The tumor cells treated with DCEVs have been shown to more effectively stimulate tumor antigen-primed T cells to secrete IFN-γ compared with untreated controls [44]. Furthermore, as tumors can disrupt antigen processing and presentation pathways in APCs, the exogenous introduction of DCEVs represents a promising strategy to overcome tumor immunosuppression. This approach has the potential to sensitize immunotherapy refractory tumors, thereby enhancing their responsiveness to immunotherapeutic treatments such as immune checkpoint blockade inhibitors [45,46].

### Specific functions of iEVs in immune synapses

The immune synapse (IS) is a highly specialized cell–cell interface established between APCs and T cells during antigen presentation. Beyond the critical receptor–ligand interactions that mediate antigen recognition and co-stimulation/co-repression, soluble cytokines and EVs have been detected in the synaptic cleft, which collectively orchestrate a robust adaptive immune response or maintain self-tolerance [47,48]. Given the minimal distance EVs need to traverse within the IS to reach recipient cells, many bioactive molecules transferred via EVs remain intact and functionally active. For instance, telomere fragments shed from APCs can be delivered to T cells through Rad51 containing EVs within the IS, where they fuse with T cell chromosome ends to elongate telomeres, thereby supporting long-term immune protection [49]. In a reciprocal manner, EVs produced by T cells can travel back to APCs, enhancing immune-stimulatory activity and establishing a positive feedback loop. Activated helper T cells, for example, secrete EVs enriched with clustered costimulatory molecules such as CD40 ligand (CD40L) and inducible T cell costimulator. These EVs promote extensive CD40 cross-linking on DC surface, leading to robust downstream DC maturation and activation [50]. Similarly, EVs released from activated CD4+ T cells, enriched with miRNAs such as miR-20, miR-25, and miR-155, can be transferred to B cells, enhancing their survival, proliferation, and antibody class switching [11]. Notably, a recent study distinguished T cell-derived EVs within the IS, also termed trans-synaptic vesicles (tSVs), from conventional T cell-derived EVs generated during *in vitro* culture, by their greater abundance of RNA-binding proteins and miRNAs [51]. This finding suggests that tSVs may possess more potent immunological activity compared with conventional EVs; however, their functional roles and mechanisms remain to be fully elucidated.

### Immune regulation

To maintain immune homeostasis and suppress excessive immune responses, the immune system employs a variety of regulatory molecular mechanisms, mainly including the expression of IC molecules and the secretion of immunosuppressive cytokines and miRNAs. When mediated via EVs, these mechanisms are likely potentiated, as the vesicular format enhances the stability and focal concentration of inhibitory molecules, amplifying their immunosuppressive effects [52].

### IC molecules

IC molecules, such as cytotoxic T-lymphocyte antigen-4 (CTLA4), programmed cell death-1 (PD-1) and its ligand PD-L1, lymphocyte activation gene 3 (LAG3), and T cell immunoreceptor with Ig and ITIM domains (TIGIT), and V-domain Ig suppressor of T cell activation (VISTA), are widely expressed on immune regulatory cells, including T regulatory cells (Treg) and myeloid-derived suppressor cells (MDSCs), to prevent excessive activation of T cells [53,54]. Among these, CTLA4 has been detected on Treg-derived EVs [55]; however, the presence of other ICs on immune regulatory cell-derived EVs remains underexplored.

To date, ICs are more frequently identified on EVs derived from tumor or stem cells. For instance, PD-L1 is enriched on breast cancer cell-derived EVs, where it binds to PD-1 on T cells, suppressing their activation and cytotoxic function, thereby dampening anti-tumor immunity within the tumor microenvironment [56]. Similarly, lung cancer cell-derived EVs carrying PD-L1 have been shown to inhibit pro-inflammatory cytokine secretion (e.g., IFN-γ) and induce CD8+ T cell apoptosis [57]. Chronic lymphocytic leukemia-derived EVs contain a plethora of ICs, such as PD-L1, TIM3 ligand, TIGIT ligand, which impair T cell viability, proliferation, activation, and metabolism, while fostering T cell exhaustion and promoting Treg differentiation [58]. Interestingly, a co-stimulatory molecule, 4–1BBL, has also been detected on leukemic EVs, where it paradoxically induces immunosuppression by expanding Tregs through modulation of mTOR and STAT5 signaling pathways [59]. In addition to tumor cells, genetically or thermally engineered mesenchymal stem cells up-regulate PD-L1 in their secreted EVs, prolonging allograft survival and offering therapeutic potential for autoimmune and chronic inflammatory diseases by promoting Treg differentiation [60-62].

Notably, IC-bearing EVs are not necessarily immunosuppressive. One example involves T cell-derived EVs that carry PD-1 on their surfaces. These PD-1–expressing iEVs can engage cell-surface PD-L1, thereby reducing the availability of PD-L1 to bind PD-1 on effector T cells. In theory, this mechanism alleviates the PD-1/PD-L1–mediated inhibition imposed by tumor cells or immunosuppressive populations, indirectly enhancing the cytotoxic and cytokine-secreting capabilities of T cells and, consequently, partially restoring anti-tumor immune function. Experimental evidence supports this model. For instance, a study by Y. Qiu et al. demonstrated that tumor-infiltrating lymphocytes release EVs carrying PD-1, which can bind PD-L1 on BT549-PD-L1 cells. This interaction triggers the internalization of PD-L1 via clathrin-mediated endocytosis, effectively reducing subsequent PD-1: PD-L1 engagement and restoring tumor surveillance [63].

### Other inhibitory molecules

Inhibitory molecules other than ICs that have also been identified on iEVs include immunosuppressive cytokines and enzymes. For instance, IL-35, a potent immunosuppressive cytokine of the IL-12 family mainly secreted by Treg, plays a critical role in infectious tolerance and the suppression of effector T cell proliferation [64]. Recent findings suggest that IL-35 is primarily delivered in the vesicular format rather than as a free cytokine, a strategy that enhances its stability. IL-35-loaded EVs coat bystander T and B lymphocytes, leading to the up-regulation of IC expression on these lymphocytes and promoting their exhaustion [65]. Treg-derived EVs also mediate immunosuppressive effects through the delivery of ectonucleotidases, such as CD39 and CD73, which hydrolyze ATP to produce immunosuppressive adenosine, priming macrophage polarization toward an anti-inflammatory phenotype [66].

Additionally, other immunosuppressive proteins, including the iNOS enzyme and neuropilin-1, have been recently reported within Treg-derived EVs [67,68]. These molecules modulate the phenotype and function of effector T cells, inhibit their proliferation, and influence macrophage differentiation. Collectively, these properties highlight the therapeutic potential of Treg-derived EVs in promoting transplantation tolerance. Besides Treg-derived EVs, cancer-associated fibroblast-induced MDSCs release EVs enriched with fructose bisphosphatase-1, a regulatory enzyme in gluconeogenesis [69]. These EVs create a tumor-permissive microenvironment by inhibiting T cell glycolysis, thereby suppressing their proliferation and function.

### Non-coding RNAs

Non-coding RNAs, particularly miRNAs, are the most extensively studied nucleic acid cargos of EVs. While effector T cell-derived miRNAs, such as aforementioned miR-25, miR-155 and miR-215, activate immune responses, specific miRNAs associated with Treg-derived EVs, such as Let-7d, miR-709, and miR-449a, reduce inflammatory response by suppressing T helper 1 cell proliferation, reducing the secretion of pro-inflammatory cytokines (e.g., IFN-γ), and targeting pro-inflammatory pathways (e.g., Notch signaling) [70-72]. Similarly, miRNAs enriched in tumor-associated macrophages (TAMs), such as miR-29 and miR-21, contribute to the establishment of an immunosuppressive microenvironment that promotes tumor progression and metastasis by increasing the abundance of Tregs and exhausted T cells while reducing T helper cells [73,74]. In addition to miRNAs, TAM-derived EVs contain long non-coding RNAs, such as NEAT1, which sponges miR-101–3p, leading to PD-L1 up-regulation on tumor cells and immune evasion [75].

Above all, continued research into the mechanisms by which inhibitory molecule-bearing EVs, particularly those derived from immune cells, mediate immune regulation and activation will be critical in unlocking their potential for the development of innovative immunotherapeutic strategies and diagnostic tools.

### The dual role of macrophage- and neutrophil-derived EVs in immune activation and regulation

Macrophages are a heterogeneous population of myeloid-lineage cells that perform diverse immunological functions in the tumor microenvironment through phenotypic polarization. Depending on their origin, macrophage-derived EVs (Mφ-EVs) play distinct roles in immune activation or regulation. M1 macrophage-derived EVs (M1-EVs) transmit pro-inflammatory signals to naive macrophages and DCs by up-regulating the expression levels of Th-1 cytokines, such as IL-6, IL-12, and IFN-γ, while suppressing IL-4 and IL-10 secretion [76]. In contrast, M2 macrophage-derived EVs (M2-EVs) exert potent anti-inflammatory effects by inducing macrophages to up-regulate the anti-inflammatory cytokine IL-10 and reprogram M1 macrophages to transform into the M2 phenotype. This immunoregulatory mechanism is critical in mitigating excessive inflammatory responses in chronic inflammatory diseases, such as osteoarthritis [77]. miRNAs are likely to be key mediators of the anti-inflammatory effects of M2-EVs. Notably, miR-709, which is abundantly present in M2-EVs, inhibits glycogen synthase kinase 3β (GSK-3β) and prevents GSK-3β-mediated degradation of β-catenin, a critical activator of the inflammation controlling Wnt/β-catenin signaling, eventually dampening inflammatory responses [78].

Similarly, neutrophils can functionally polarize into pro-inflammatory N1 and pro-regenerative N2 subtypes, releasing distinct EVs (N1EVs and N2EVs) with multiple functions. N1EVs are enriched with inflammation-related and cytotoxic molecules that promote macrophage activation, T cell proliferation, and induce tumor cell apoptosis through the activation of caspase signaling pathways. In contrast, N2EVs carry regulatory molecules that facilitate tumor growth and survival by reactivating tumor cells, angiogenesis, and immune evasion via induction of M2 macrophage polarization [79]. In summary, the macrophage- or neutrophil-derived EVs (NEVs) exhibit complex and sometimes controversial functions; however, they play a critical role in maintaining the balance between pro-inflammatory and anti-inflammatory signaling [80].

### Engineered iEVs-mediated immune modulation

In addition to naturally occurring EVs, artificially modified EVs can enable more precise and efficient immune modulation. These engineered EVs can be generated through two main approaches. The first involves post-secretion engineering of EVs, such as fusing them with synthetic nanoparticles to confer multifunctionality and enforcing exogenous proteins or nucleic acids to be loaded into EVs or onto their membrane surface via electroporation or membrane fusion. The second strategy is to engineer the parent cells to produce EVs that inherently carry specific membrane proteins or bioactive cargo. Both approaches yield EVs with tailored functionalities, offering a more targeted and effective platform for therapeutic applications.

In post-secretion engineering, NEVs have been widely utilized to enhance targeted delivery of nanoparticles, leveraging their intrinsic ability to preferentially accumulate at inflammatory sites. For instance, neutrophil-derived exosomes were functionalized with ultra-small Prussian blue nanoparticles (uPB, < 5 nm) by click chemistry, resulting in uPB-Exo [81]. This engineered construct retained the innate targeting properties of NEVs while exhibiting potent anti-inflammatory effects. Specifically, uPB-Exo selectively accumulated in activated fibroblast-like synoviocytes, neutralized pro-inflammatory factors, scavenged reactive oxygen species, and alleviated inflammatory stress. Furthermore, they penetrated cartilage, facilitating real-time visualization and precise diagnosis of rheumatoid arthritis through the magnetic resonance imaging. Similarly, NEV-like nanovesicles modified with superparamagnetic iron oxide nanoparticles have demonstrated dual biological and magnetic targeting capabilities, enabling selective accumulation at tumor sites and enhanced tumor cell inhibition when further loaded with doxorubicin [80]. Beyond neutrophils, DCEVs exhibit a high capacity for antigen peptide loading via peptide pulsing, presumably attributable to their high surface expression of MHC molecules [82]. In a clinical study, tumor antigen-loaded DCEVs effectively induced a Th1-polarized immune response, stimulated interferon secretion, and promoted the proliferation of cytotoxic T lymphocytes in vivo [83]. Electroporation is a widely used technique for loading nucleic acids into EVs. For example, siRNA targeting β-site amyloid precursor protein cleaving enzyme 1 (BACE1), a key enzyme in amyloid-β peptide production, was successfully introduced into DCEVs via electroporation without their structural integrity. The resulting BACE1 siRNA-loaded EVs effectively down-regulated BACE1 mRNA in the mouse brain and alleviated Alzheimer’s disease symptoms [82].

Genetic engineering of parental cells prior EV secretion has been exemplified by CAR-T cell-derived EVs. These EVs carry the CAR construct on their surface, are enriched with cytotoxic molecules, but devoid of checkpoint molecules like PD-1, thereby facilitating more potent and direct tumor cell eradication compared with EVs secreted by natural T cells. Compared with CAR-T cell therapy, CAR-T cell-derived EVs may offer enhanced safety by avoiding uncontrolled T cell expansion and excessive cytokine production [84]. Similarly, CAR-NK cells generate EVs (CAR-NK-EVs) displaying DR5-agonistic single-chain variable fragments (scFv), which specifically bind to DR5+ tumor cells, MDSCs, and cancer-associated fibroblasts, and trigger their apoptosis. Beyond relieving immune suppression, DR5-scFv + CAR-NK-EVs have been shown to activate CD8+ T cells in organotypic melanoma slices obtained from patients [85]. Moreover, these EVs have demonstrated significant tumor growth inhibition and extended survival in multiple preclinical tumor models, including melanoma, liver cancer, and breast cancer, outperforming anti-DR5 antibodies. Other parental cells can also be genetically engineered to enhance EV functionality. For instance, DCs can be transfected with a plasmid encoding Lamp2b fused with a targeting peptide, resulting in the production of EVs displaying this peptide for targeted delivery [82]. Additionally, parental DCs can be engineered to express immunostimulatory or cytotoxic molecules, such as TNF or TNF-related apoptosis-inducing ligand, which can be transmitted to EVs, enhancing intrinsic antigen presentation and immune activation effects of natural DCEVs.

In addition to immune activation, genetic modification of immune cells to introduce or up-regulate specific immunomodulatory factors can enhance the immunoregulatory capabilities of their EVs. For example, bone marrow stromal cells (BMSCs) engineered to overexpress TIM3 produced EVs that, when incorporated into a hydrogel for controlled release, promoted M2 macrophage polarization, suppressed the p38/MAPK pathway, and alleviated excessive inflammation, ultimately increasing BMP2 secretion and improving bone integration [86]. Similarly, EVs derived from parental bone marrow dendritic cells (BMDCs) transfected with a IL-10-expressing adenovirus inhibited delayed-type hypersensitivity (DTH) in mice, highlighting their immunosuppressive potential [86]. Engineered iEVs also hold promise in IC blockade therapy. In a recent study, DCs were genetically engineered to co-express an anti-CD19 scFv and PD1, producing bispecific EVs (bisEVs), capable of simultaneously targeting tumor antigens and blocking PD-L1, thereby reshaping the immune environment within solid tumors. Advances in genetic engineering have thus expanded the range of iEV applications, offering innovative tools for immune modulation [87].

## Challenges and future prospects in clinical applications

EVs have emerged as promising tools in various clinical applications, including biomarker discovery, drug delivery, and EV-based therapies and vaccines. Currently, EVs extracted from specific tissues or bodily fluids, such as plasma and urine, have been extensively studied using multi-omics approaches as biomarkers for disease detection and progression monitoring [88]. In therapeutic applications in translational research, EVs are mainly sourced from bone marrow mesenchymal stem cells (MSCs) with a lot of ongoing studies evaluating the efficacy of MSC-derived EVs in ophthalmic diseases and diabetes-related conditions.

Notably, iEVs, particularly those from peripheral blood cells, have elicited increased interest over the past five years for developing novel therapeutic strategies in infectious diseases and immune-related disorders. For instance, a clinical trial registered on ClinicalTrials.gov (NCT04389385) studied the use of inhaled allogeneic T cell-derived EVs in early-stage COVID-19. However, despite advancements in immune cell therapies, translating iEVs into clinical application remains limited by technical challenges.

A major limitation is the batch-to-batch variability and poor yield, which depend heavily on the phenotype and other characteristics of their parent cells and culture conditions. Factors such as cell concentration, nutrient availability, and cytokine supply can influence EV cargo composition. EV production is typically low, with less than 1 μg of EV protein generated from 1 ml of culture medium, whereas most studies require effective doses exceeding 10 μg. This limitation is particularly pronounced for short-lived immune cells; for instance, neutrophils have an *in vitro* lifespan of less than 24 hours, severely restricting their EV production and impeding pre-clinical functional validations. EV manufacturing and quality control pose additional hurdles for clinical applications [80]. Large-scale production must comply with Good Manufacturing Practice (GMP) regulations, requiring strict isolation, purification, characterization, and quality assessment to ensure the consistency of EV physical properties and biological activity [89]. Some studies have demonstrated the feasibility of monocyte-derived DCs for EV production under GMP guidelines, yet further optimization is necessary [90]. Additionally, the high cost and complex procedures involved in isolating immune cells from human peripheral blood further complicate the large-scale industrialization of iEVs.

To overcome these challenges, researchers are actively developing more efficient EV collection and purification approaches, including density gradient centrifugation, size exclusion chromatography, polymer-based precipitation, and immunoaffinity capture, to improve EV yield, quality, and batch-to-batch uniformity, thereby enhancing their scalability and clinical feasibility [91]. Another critical approach is the precise targeting of EVs to specific tissues or cells, ensuring efficient delivery while minimizing off-targeting side effects and reducing the required therapeutic dose. Despite these obstacles, iEVs hold significant clinical potential. With continued advancements in bioengineering, nanotechnology, and synthetic biology, future research will likely focus on optimizing iEV yield and functional engineering, expanding their potential as novel immunotherapies for cancer, autoimmune and inflammatory diseases, and infectious diseases.

## Conclusions

**Figure 1:**
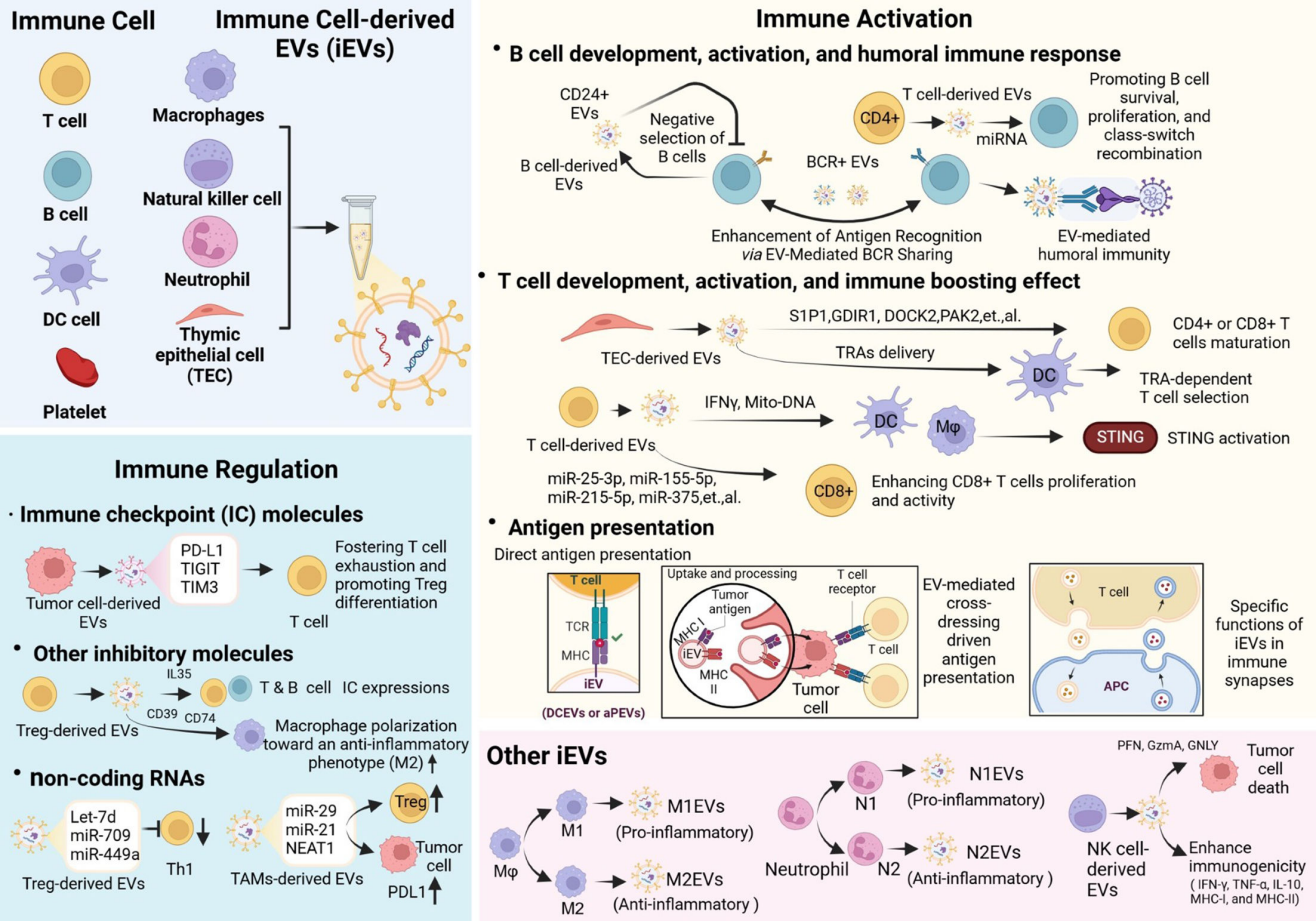
Schematic illustration of the origins, functions, and mechanisms of iEVs in immune activation and regulation. (Created in BioRender. https://BioRender.com/y54v782).

The immune system operates through a complex network of diverse cell subtypes that release stimulatory and inhibitory signals to maintain homeostasis and defend against endogenous or exogenous insults. As a pivotal intercellular communication mediator, EVs have recently emerged as a key player in immune activation and regulation. In this review, we have summarized recent advancements in understanding how iEVs and their associated molecules contribute to the development, activation, and regulation of the immune system (Figure 1). We also discussed the multifaceted immunological functions of EVs derived from different immune cell types. For example, EVs from macrophages and neutrophils exhibit dual roles in immune activation and suppression, depending on their polarization states.

To date, most studies in this field have focused on elucidating the mechanisms of iEVs and assessing their therapeutic potential in animal models. In particular, engineering parental immune cells to enrich functional molecules within EVs has shown great promise. In this review, we systematically summarize the disease-specific applications of iEVs derived from various parental cells and generated using diverse engineering strategies (Table 1). Engineered iEVs can be obtained either through post-secretion modification or parental cell manipulation, enabling more precise immunomodulation with enhanced targeting capacity, immune activation, or immunosuppressive effects for treating cancer, autoimmune diseases, and inflammatory conditions.

**Table 1:** iEV engineering approaches and their therapeutic applications in various diseases.

Cell type	Engineering method	Disease type/Application
Neutrophils	Post-secretion followed by functionalization with ultra-small Prussian blue nanoparticles (uPB) via click chemistry [81]	Rheumatoid arthritis (enhanced targeting of inflamed synovium, anti-inflammatory effects, MRI-based real-time visualization)
	Post-secretion followed by functionalization with SPIONs [80]	Cancer therapy (dual biological and magnetic targeting, tumor-selective accumulation, enhanced tumor cell inhibition with doxorubicin)
DCs	Post-secretion followed by peptide pulsing (high MHC expression facilitates peptide loading) [83]	Cancer immunotherapy (Th1-polarized immune response, IFN secretion, cytotoxic T-lymphocyte proliferation)
	Post-secretion followed by BACE1 siRNA loading via electroporation [82]	Alzheimer’s disease (reduction in BACE1 mRNA in the brain and symptomatic relief)
	Genetic engineering of parental cells with a Lamp2b-targeting peptide fusion plasmid [82]	Targeted delivery (EVs displaying specific peptides on their surface)
	Genetic engineering of parental cells with IL-10-expressing adenovirus [86]	Hypersensitivity (immunosuppression)
	Genetic engineering of parental cells to co-express anti-CD19 scFv and PD1 (bispecific EVs) [87]	Cancer immunotherapy (immune checkpoint blockade and tumor targeting)
BMSCs	Genetic engineering of parental cells to overexpress TIM3 [86]	Bone repair and immunomodulation (M2 macrophage polarization, p38/MAPK suppression, increased BMP2 secretion)
T cells (CAR-T cells)	Genetic engineering of parental cells with CAR construct [84]	Cancer immunotherapy (EVs carry CAR on surface, enriched with cytotoxic molecules, lack PD-1 for potent tumor cell eradication, improved safety)
NK cells (CAR-NK cells)	Genetic engineering of parental cells to express DR5-agonistic scFv [85]	Cancer immunotherapy (melanoma, liver cancer, breast cancer; triggers apoptosis in DR5+ tumor cells, MDSCs, CAFs, and activates CD8+ T cells)

BMSCs, bone marrow stromal cells. DCs, dendritic cells. EVs, extracellular vesicles. iEV, immune cell-derived EVs. MHC, major histocompatibility complex. NK, natural killer. SPION, superparamagnetic iron oxide nanoparticle.

Despite their considerable potential in disease diagnosis, drug delivery, and immunotherapy, the clinical translation of iEVs still faces several challenges. These include low yield, batch-to-batch variability, limited scalability due to intrinsic immune cell characteristics, and complex manufacturing processes required to meet GMP standards. Additionally, the high cost and technical difficulty of isolating immune cells further hinder large-scale industrial applications. To overcome these limitations, researchers are actively developing more efficient strategies for EV purification and targeted delivery. With continued advances in bioengineering, nanotechnology, and synthetic biology, iEVs are poised to become powerful therapeutic tools for cancer, autoimmune, inflammatory, and infectious diseases.

**Summary**

- Immune cell-derived extracellular vesicles (iEVs) serve as essential mediators of intercellular communication, potentially activating and modulating adaptive and innate immunity by stimulating or suppressing immune cell development and activity, regulating cytokine production, and influencing antigen presentation.
- In immune activation, iEVs derived from T, B, and natural killer cells promote immune cell development and activation, thereby enhancing immune function. During antigen presentation, iEVs contribute via direct interaction (acting like APC cells) and cross-dressing (transferring MHC-antigen complexes). Furthermore, iEVs support adaptive immune responses and intercellular communication by delivering bioactive molecules within immune synapses. In immune regulation, the role of immune checkpoint molecules carried by iEVs is controversial: while they enhance immunosuppressive functions in tumors and regulatory immune cells, they may also counteract PD-1/PD-L1 inhibition to improve anti-tumor immunity. Other immune regulatory molecules, such as IL-35, CD39, and non-coding RNAs, are delivered by iEVs, which increase their stability and enhance their immunosuppressive activities.
- Macrophage- and neutrophil-derived EVs exhibit dual roles in immune activation and regulation by promoting either pro-inflammatory or anti-inflammatory responses depending on their polarization state.
- Engineered iEVs enhance immune modulation through post-secretion or parental cell engineering strategies, enabling precise delivery, immune activation, and immunosuppression for diverse therapeutic applications.
- Despite challenges in yield, consistency, and large-scale production, iEVs hold great clinical potential as next-generation therapies (e.g., vaccine development and serving as a drug delivery vehicle), and as biomarkers for diagnosing immune-related diseases and evaluating immunotherapy outcomes. Advances in bioengineering and nanotechnology are expected to enhance their scalability, targeting specificity, and therapeutic efficacy.

## Abbreviations

BMSCs: bone marrow stromal cells
DCEVs: DC-derived EVs
DCs: dendritic cells
EVs: extracellular vesicles
GMP: Good Manufacturing Practice
MHC: major histocompatibility complex
NK: natural killer
NKEVs: NK cell-derived EVs
aPEVs: activated platelet-derived EVs
iEVs: immune cell-derived EVs
tRFs: tRNA fragments
